# Noninvasive imaging of tumor hypoxia after nanoparticle-mediated tumor vascular disruption

**DOI:** 10.1371/journal.pone.0236245

**Published:** 2020-07-24

**Authors:** Needa A. Virani, Olivia J. Kelada, Sijumon Kunjachan, Alexandre Detappe, Jihun Kwon, Jennifer Hayashi, Ana Vazquez-Pagan, Douglas E. Biancur, Thomas Ireland, Rajiv Kumar, Srinivas Sridhar, G. Mike Makrigiorgos, Ross I. Berbeco

**Affiliations:** 1 Department of Radiation Oncology, Brigham and Women’s Hospital, Dana-Farber Cancer Institute and Harvard Medical School, Boston, Massachusetts, United States of America; 2 Department of Medical Oncology, Dana-Farber Cancer Institute and Harvard Medical School, Boston Massachusetts, United States of America; 3 Department of Radiation Oncology, Hokkaido University, Sapporo, Japan; 4 Nanomedicine Innovation Center and Department of Physics, Northeastern University, Boston, Massachusetts, United States of America; 5 LA-ICP-MS and ICP-ES Laboratories, Boston University, Boston, Massachusetts, United States of America; Northwestern University Feinberg School of Medicine, UNITED STATES

## Abstract

We have previously demonstrated that endothelial targeting of gold nanoparticles followed by external beam irradiation can cause specific tumor vascular disruption in mouse models of cancer. The induced vascular damage may lead to changes in tumor physiology, including tumor hypoxia, thereby compromising future therapeutic interventions. In this study, we investigate the dynamic changes in tumor hypoxia mediated by targeted gold nanoparticles and clinical radiation therapy (RT). By using noninvasive whole-body fluorescence imaging, tumor hypoxia was measured at baseline, on day 2 and day 13, post-tumor vascular disruption. A 2.5-fold increase (*P<0*.*05*) in tumor hypoxia was measured two days after combined therapy, resolving by day 13. In addition, the combination of vascular-targeted gold nanoparticles and radiation therapy resulted in a significant (*P<0*.*05*) suppression of tumor growth. This is the first study to demonstrate the tumor hypoxic physiological response and recovery after delivery of vascular-targeted gold nanoparticles followed by clinical radiation therapy in a human non-small cell lung cancer athymic *Foxn1*^*nu*^ mouse model.

## Introduction

A key component propagating tumor growth is the presence of high vascular infiltration and oxygen diffusion [1, 2]. As tumors progress beyond a few millimeters in size, rate of cell proliferation exceeds the rate of vasculature neoangiogenesis leading to areas of limited oxygen and nutrient supply. Due to the rapid neovascular growth rate, vessels are usually chaotic and abnormal with large pores allowing for passive accumulation of small molecules and nanoparticles [3–5]. The importance of the vascular supply for continued tumor growth makes it a potential therapeutic target for both radiation and chemotherapy [6, 7].

Vascular targeting agents can either a) inhibit angiogenesis to control the development of blood vessels [6, 8], or b) restrict the function of existing blood vessels using vascular disrupting agents (VDAs) [5, 9]. Multiple small molecule VDAs have been tested in clinical trials with limited success in translation [9–12]. A key downfall of VDA treatment alone is the remaining viable rim of tumor cells surrounding the necrotic, ischemic treated region leading to continued tumor growth[5, 9]. Although some of these vascular targeting agents have shown to be effective when used in conjunction with conventional therapies, such as external beam radiation therapy, they have caused severe off-target toxicity [7, 8, 10–16]. In this work, we investigate vascular-targeted gold nanoparticles, which act as VDAs when irradiated after delivery. Combining a targeted VDA concept with the precision of modern image-guided radiation therapy enables double targeting and limits side effects by avoiding the activation of any gold nanoparticles that may accumulate in surrounding healthy tissue. This dual targeting strategy can minimize normal tissue toxicity and consequently improve the therapeutic benefit [17–20].

Metallic nanoparticles have been developed for use in combination with radiation therapy to intensify the damage to tumor cells *via* radiation dose amplification. This is due to the physical interaction of low energy x-rays with high-Z elements resulting in the emission of both Auger electrons and short-range photoelectrons leading to local cell damage [21–24]. Gold nanoparticles (AuNP) are of particular interest as radiosensitizers due to their biocompatibility. Gold nanoparticles have a high K-edge (≈81 keV), which enables local, controlled dose enhancement when coupled with conventional radiation therapy [25–27]. The ease of surface modification of nanoparticles and targeting capabilities to neovascular regions offers new possibilities for anti-vascular therapies.

Theoretical and experimental studies have shown that gold nanoparticles can impart considerable dose amplification to endothelial cells even without specific cellular uptake [18, 22, 28, 29]. Experimentally, we have previously shown that Arginylglycylaspartic acid (RGD) peptide surface modified gold nanoparticles boost local radiation doses due to the generation of short-range electrons resulting in tumor blood vessel disruption [30] and subsequent changes in tumor physiology indicative of a propensity for improved drug delivery [31]. RGD peptide has a strong affinity for αvβ3 and can be used as a tumor vasculature specific targeting agent. RGD is involved in protein and cell attachment, making it a prime candidate for the delivery of vasculature specific nanoparticles [30, 32].

While efficacy has been previously demonstrated, there is a lingering concern that vascular disruption therapy could lead to the development of hypoxic regions within the tumor where the high rate of oxygen demanded and consumed by the tumor and endothelial cells exceeds supply [33, 34]. The hypoxic tumor microenvironment hinders response to chemotherapy and radiation treatment resulting in an overall reduction in survival due to the reduction in cellular oxygenation [35]. Hypoxia further stimulates inducible factors leading to increased tumor proliferation (tumor invasion and metastases *via* tumor neovasculature) and a more aggressive phenotype [35–37]. The challenge of tumor hypoxia in cancer therapy has been known for over 60 years and is still a significant clinical problem today [38].

In the study presented, we investigated the impact of tumor vascular disruption on tumor hypoxia as the latter can lead to radiation resistance and is associated with a worse prognosis. To this end, we used vascular-targeted AuNP combined with clinical radiation (10Gy, 6MV) and performed longitudinal *in vivo* imaging of tumor hypoxia. Tumor regression studies were also performed on pre-clinical mouse models bearing human non-small cell lung cancer (NSCLC, A549) to investigate the potential therapeutic benefit of this dual-targeting approach.

## Materials and methods

### Animal tumor model and *in vivo* studies

Animal studies were approved by the Dana Farber Cancer Institute Institutional Animal Care and Use Committee (DFCI IACUC, Protocol Number 14–032) and conducted in full compliance with the Association for the Assessment and Accreditation of Laboratory Animal Care, governmental and institutional regulations and principles outlined in the United States Public Health Service Guide. 4–6 week old female athymic *Foxn1*^*nu*^ mice (Charles River Laboratories, Wilmington, USA) were used. Mice weighing ~25 g were fed *ad libitum* with standard food pellets and water. Human lung adenocarcinoma cells, A549 (ATCC CCL-185, Manassas, VA), were grown *in vitro* in Roswell Park Memorial Institute medium (RPMI 1640; Gibco, Invitrogen, USA), supplemented with 10% fetal bovine serum (FBS) (Invitrogen, USA) and 1% pen/strep (10,000 U/mL penicillin; 10,000 μg/mL streptomycin, Invitrogen, USA). The culture was maintained at 37°C, 5% CO_2_ and optimal humidity. Mice were inoculated with 3x10^6^ cells/100 μL by subcutaneous injection into the dorsolateral left flank. Tumor volume was determined with calipers using the formula: volume = (length × width^2^)/2 and a tumor size of ≈ 8 mm^2^ was obtained in ~2 weeks. Mice were euthanized when tumors were more than 10% of animal body weight or if any other signs of distress were identified (ulcerations, lethargy, etc).

### Chemical synthesis and characterization of functionalized targeted gold nanoparticles for tumor vascular targeting

Standard procedures were used to prepare targeted gold nanoparticles and are described in greater detail elsewhere [39]. Colloidal gold was prepared by the reduction of chloroauric acid in the presence of a stabilizing/reducing agent, tetrakis(hydroxymethyl) phosphonium chloride (THPC). The PEGylation of THPC stabilized gold nanoparticles was carried out by ligand exchange process using optimized ratios of three heterobifunctional PEGs (Thiol-PEG-Amine, Thiol-PEG-Carboxyl and Thiol-PEG-methoxy). The functionalized gold nanoparticles were further conjugated with Arginylglycylaspartic acid (RGD) peptide to the terminal carboxylic (−COOH) groups via EDC chemistry for vascular targeting and a near infrared dye (AF647) was attached for imaging. Subsequently, the resultant product, PEG-RGD-AuNP-AF647 (for the remainder of this study will be referred to as AuNP), was further purified and characterized. Nanoparticles were purified using a membrane-filtration technique. AuNP was subjected to membrane dialysis by using ≈ 14 kDa cellulose membrane against purified double-distilled water to remove any traces of un-reacted EDC or other hydrolyzed products along with the un-reacted RGD molecules resulting in a final solution comprised of purified AuNP.

### AuNP biodistribution

Mice were sacrificed at 1 h, 4 h, 12 h, 24 h, and 30 day time points post-intravenous (i.v.) injection of ~1 mg/g of AuNP (as per previous dosing studies [30]) and the tumor and other vital organs were excised. Biodistribution of AuNP was measured by inductively coupled plasma mass spectrometry (IC-PMS) for determination of the optimal therapeutic time point for radiation delivery based on the accumulation of AuNP in the tumor and other organs. All samples were frozen with liquid nitrogen and then ground in an agate mortar and pestle. ~0.03 g of sample was weighed out and digested in 3 mL HCl and 1 mL HNO3 on a hotplate at 110˚C overnight. Once removed from the hot plate, 1 mL H_2_O_2_ was added slowly and completed in two 0.5 mL steps. Samples were dried on a hot plate overnight before adding 0.25 HCl. Then 0.3 mL HCl was added and allowed to sit overnight followed by 0.25 mL H2O2. Samples were then diluted with MQ water to reach a 3% HCl concentration solution. Just prior to ICP-MS, samples were diluted further 100X. Standards were prepared using a pure Au standard and diluting with 3% HCl. Standards were made at concentrations of 0.1, 0.25, 0.5, 0.75, 1, 2.5, 5, 10 and 20 ppb.

### Transmission electron microscopy imaging

High-resolution transmission electron microscopy (TEMJEOL 2010F, Philips Netherlands) was performed on cells and tumor samples. A549 cells were imaged 2 hours after incubation with 0.1 mg/mL AuNP. Afterward, samples were placed at 4°C in dark conditions. High-resolution transmission electron microscopy was carried at magnifications of 30000x to visualize uptake of the AuNP. For *in vivo* samples, TEM imaging was performed on A549 lung tumor sections. Small tumor tissue fragments/ pieces (≈1–2 mm^3^) were obtained from the dissected whole tumor and fixed using a mixture of 2.5% glutaraldehyde in 0.2 M Sorensen buffer at pH 7. Using ultracryotome, thin sections were sliced and washed/stained in aqueous uranyl acetate (2%) for ≈2 h. The tissue was then placed at 4°C in dark conditions and further dehydrated by 100% ethanol and propylene oxide to be embedded in liquid epoxy resin (Epon™). High-resolution electron microscopy was carried out at different magnifications ranging from 2500x - 10000x to visualize the accumulation of AuNP in tumor blood vessels 24 h post-AuNP injection of 1mg/g AuNP.

### Whole body hypoxia imaging

Fluorescence imaging was performed on animals using the IVIS system (PerkinElmer, Boston, MA) prior to irradiation (0 h), as well as post-irradiation with a 6MV clinical beam (48 h and 13 days) after injection of a hypoxia-specific fluorophore marker (HypoxiSense680). Groups such as control (no AuNP / no IR), AuNP only, IR only and AuNP+IR were used for the studies and imaged for longitudinal changes in tumor hypoxia.

The Hypoxisense680 (HS680) imaging probe was administered as per the manufacturer’s protocol (PerkinElmer, Boston, MA). Each mouse was intravenously injected with 2 nmol (100 μL) of the reconstituted imaging probe 48 h prior to fluorescence imaging (FLI). For each imaging session, mice were anesthetized with an isoflurane/air mixture gas and positioned prone in the imaging cassette which was then placed into the imaging chamber (Em = 720 nm, Ex = 675 nm). The selection of excitation/emission wavelengths was chosen to effectively minimize the AF647 signal as seen in a representative excitation/emission spectra in S1 Fig. Autofluorescence background was subtracted by determining the mean tumor fluorescence signal in normalized counts in the image prior to injection. The collected fluorescence data were analyzed using Living Image Software (version 4.2). Tumor regions of interest (ROI) were drawn, and a threshold was applied to all animals (equal to 30% of the mean tumor fluorescence of positive control mice) to calculate the hypoxic fraction of the tumors.

### Clinical external beam radiation therapy

Irradiations were performed 24 h after baseline tumor hypoxia imaging and injection of AuNP with a 6MV linear accelerator (TrueBeam, Varian Medical Systems, Palo Alto, CA). For clinical beam radiations, A549 tumor bearing mice in the IR and AuNP+IR groups were anesthetized with a mix of ketamine/xylazine (10:1) before irradiation. Eclipse (Varian Medical Systems, Palo Alto, CA) clinical treatment planning system was used to calculate the tumor dose distribution using the analytical anisotropic algorithm (AAA) for a 5 x 5 cm^2^ field size at a gantry angle of 180 degrees, SSD of 90 cm, dose rate of 600 MU/min and a prescription of 10 Gy to the center of the tumor. A 10-cm depth for the tumor was created with solid water (CIRS, Norfolk, VA). Normal tissue was shielded by the positioning of the primary collimator as previously described [23, 40].

### Tumor regression and time-to-tumor-doubling study

Four groups (Control (-AuNP/-IR); AuNP only, IR only, and AuNP+IR) of 9–10 mice each were monitored for changes in tumor size for up to 50 days following tumor inoculation. Tumor response was measured by calipers using the formula: volume = (length × width^2^)/2. Mouse body weight was recorded, and behavior was monitored throughout the experiment to assess systemic toxicity. Mice were euthanized when tumors were more than 10% of animal body weight or if any other signs of distress were identified (ulcerations, lethargy, etc). Time-to-Tumor-Doubling (TTD) was defined as time for tumor volume to double relative to day of treatment.

### Tumor hypoxia histological immunohistochemistry

Histological examinations were carried out to investigate the effect of nanoparticles and 6MV clinical beam treatment (+/-AuNP; +/-IR) on tumor hypoxia. For tumor hypoxia staining, pimonidazole (Hypoxyprobe, Burlington, MA) was dissolved in neutral buffered saline and administrated *i*.*v*.at a concentration of 60 mg/kg body weight with mice sacrificed 90 min later. The excised tumor tissues were fixed in 10% formalin (neutral buffered) and embedded in paraffin. Thin tissue slices (≈5μm) were cut, and the sections were mounted for antigen retrieval. Standard IHC steps of de-paraffinizing and rehydrating was followed by staining with the mouse mAb FITC-labeled anti-pimonidazole antibody (Hypoxyprobe, Burlington, MA) and incubating for 1 h at 37 ˚C in the dark as per the manufacturer's protocol. Following blocking and DAB steps, images were visualized using a Leica DMi8 (Leica Microsystems, Germany) widefield microscope with a high-resolution AxiocamMrm Rev.3 camera at 20x magnification.

### Toxicity histology

For the assessment of treatment toxicity (+/-AuNP; +/-IR), mice bearing A549 subcutaneous tumors were injected with either saline or ~1 mg/g AuNP followed by 10 Gy radiation with a 220 kVp Small Animal Radiation Research Platform (SARRP, Xstrahl, Suwanee, GA) after 24 h. SARRP radiations were performed similar to previous studies [30]. Mice were then euthanized and vital organs (liver, lungs, spleen, kidney, and heart) were collected for histology 24 h post-treatment. H&E histological staining was carried out on all excised tissues.

### Statistical analysis

Biodistribution and Hypoxisense statistical significance was evaluated using a standard student’s unpaired two-tailed *t*-test *(P<0*.*05)*. Relative tumor volume statistical analysis was performed using Mann-Whitney nonparametric U test *(P < 0*.*05)* in GraphPad Prism (version 8). TTD statistical analysis was performed using a Log-Rank (Mantel-Cox) test *(P < 0*.*05)* in GraphPad Prism (version 8).

### Data availability

All relevant data are within the manuscript.

## Results

**Fig 1:**
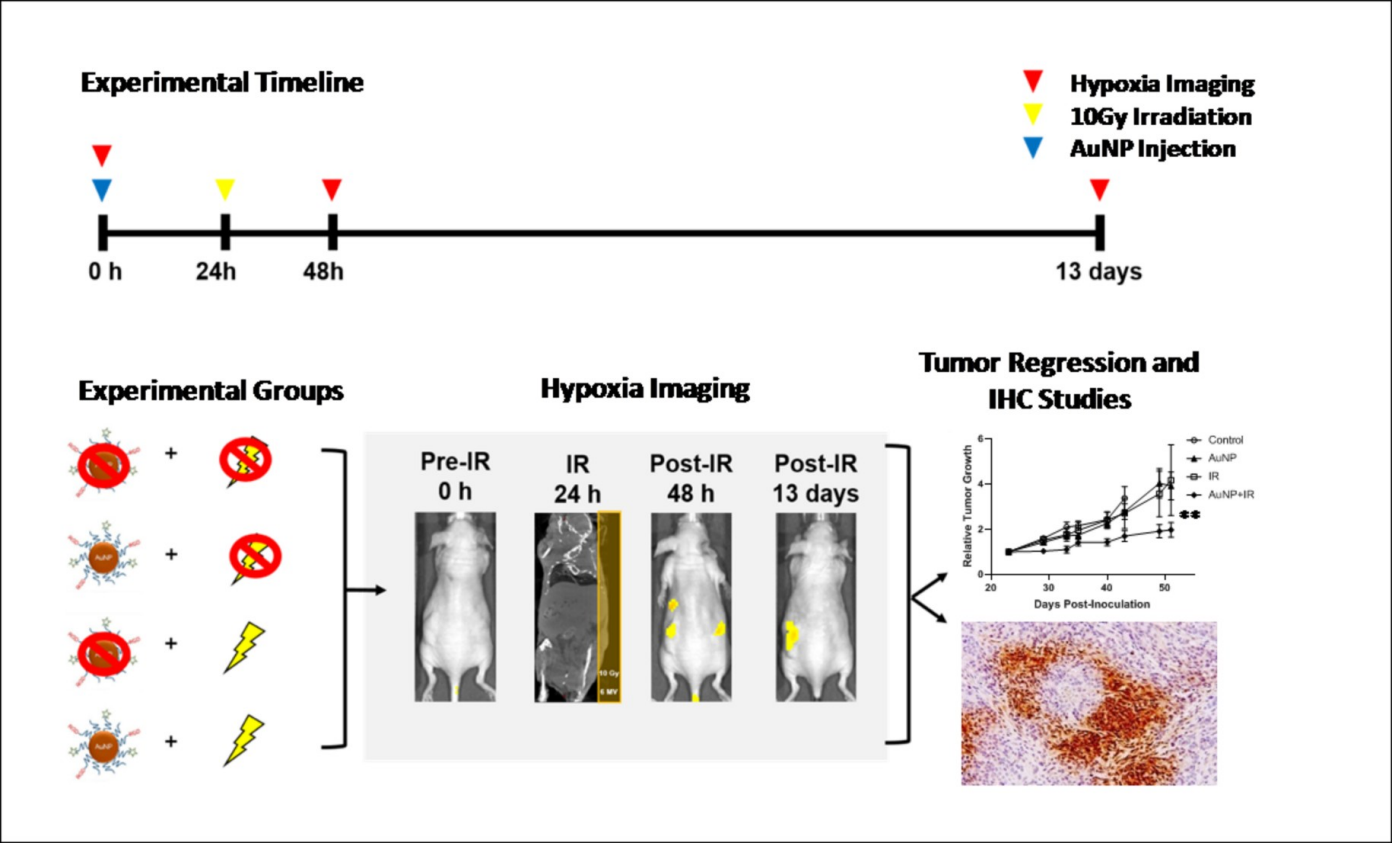
Experimental timeline and design. A schematic depiction of the experimental design to measure tumor hypoxia after radiation-induced tumor vascular damage is shown. In a human NSCLC xenograft model (A549) gold nanoparticles (AuNP, blue arrow) were *i*.*v*. administered to target tumor neovessels, and 10 Gy radiation was delivered (yellow arrow) 24 h post-AuNP injection. HypoxiSense680 fluorescence imaging (red arrow) was used to assess changes in tumor hypoxia pre (0 h) and post-treatment (48 h and 13 days) for all four groups (Control, AuNP, IR, and AuNP+IR). After imaging, mice were a) monitored for tumor progression or b) euthanized for immunohistochemistry studies to confirm tumor hypoxia.

The conceptual design and the experimental set-up is depicted in Fig 1. Targeted gold nanoparticles were co-functionalized with polyethylene glycol (PEG) and RGD. PEGylation provides extended systemic circulation to allow AuNP to reach the tumor and RGD confers tumor vascular targeting *in vivo* [39]. High-resolution transmission electron microscopy (TEM) imaging showed AuNP to have spherical surface morphology with an average AuNP core diameter of~3–4 nm and DLS determined the hydrodynamic size to be ~10–12 nm, an appropriate size for renal clearance [41]. The absorption and fluorescence spectra of AuNP was λmax of 650/668 nm post-labeling respectively and the average zeta-potential (surface charge) was +7.93 mV in PBS (7.4 pH) [39].

**Fig 2:**
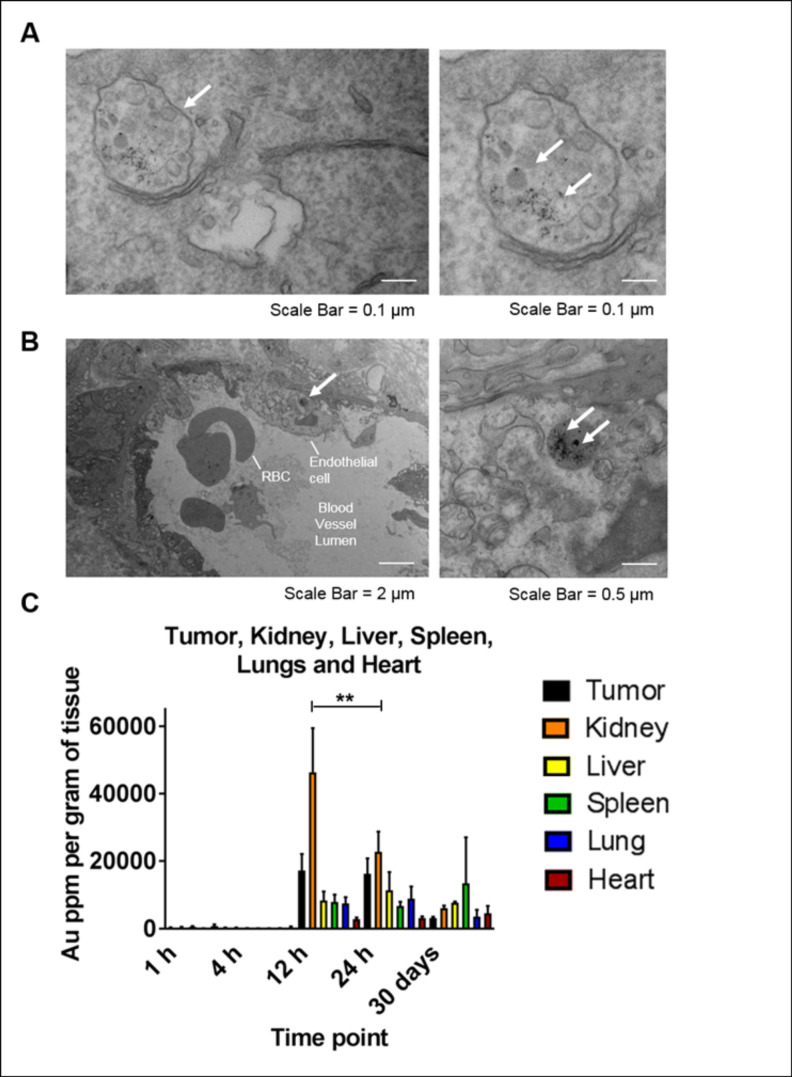
Tumor uptake and biodistribution studies. (A) Tumor uptake of AuNP at 2 h post-incubation in A549 cells was measured using high-resolution TEM. Localized uptake in several sub-cellular compartments was observed following 2 h incubation. (B) High-resolution transmission electron microscopy was carried out on *ex vivo* A549 lung tumor sections 24 h after 1mg/g AuNP injection. White arrows indicate tumor cell/vasculature uptake of AuNP (at a magnification of 2500 - 10000x) and labels indicate the location of red blood cells and endothelial cells. (C) Longitudinal accumulation of AuNP in the tumor, kidney, liver, spleen, lungs, and heart was measured at 1, 4, 12, 24 h, and 30 days after *i*.*v*. administration of ~1mg/g of AuNP (n = 2–5). The amount of Au was quantified using ICP-MS and normalized with respective organ weights. Values represent mean ± SD, (**P<0.*05*).

**Fig 3:**
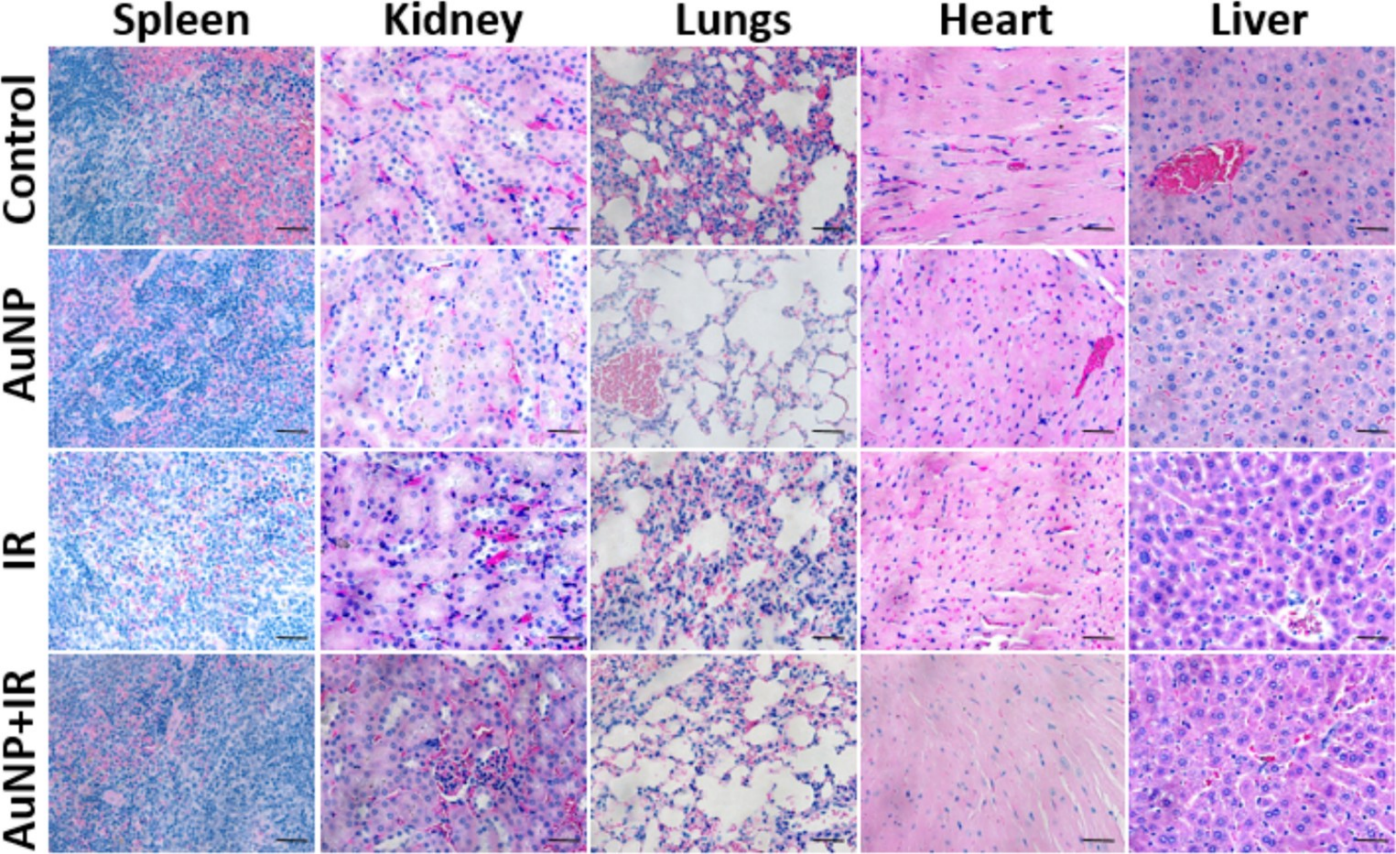
Evaluation of treatment toxicity. H&E staining indicated no detectable toxicity in surrounding visceral organs such as spleen, kidney, lungs, heart, and liver due to either AuNP and/or radiation treatment. Scale bar: 50 μm.

The deposition and early uptake of AuNP clusters were observed in the A549 tumor cell vesicles *in vitro* after 2 h of incubation with 0.1 mg/mL AuNP (Fig 2A). Uptake in the tumor and tumor vasculature at 24 h post-AuNP intravenous injection were also visualized in TEM imaging (Fig 2B) to verify nanoparticles have accumulated within the tumor and tumor vasculature at time of radiation delivery. In Fig 2C, biodistribution studies confirmed maximum tumor accumulation of AuNP at 12 and 24 h, with no statistically significant difference between the time points. However, at 24 h significant (*P<0*.*05*) clearance of AuNP from the kidney was observed compared to 12 h, while accumulation in all other organs (liver, spleen, lungs, and heart) was not substantially different. Previous literature indicates that AuNPs are rapidly eliminated from the body *via* renal clearance [42, 43]. Other studies have also shown that the size, composition, shape, and surface charge of AuNP nanoparticles may also direct clearance via the hepatobiliary pathway[44, 45]. Evaluation of treatment toxicity was assessed using H&E histological staining (Fig 3). At 24 h post treatment, H&E staining indicated no detectable toxicity or morphological changes to the surrounding organs (liver, lungs, spleen, kidney, and heart) due to either AuNP and/or radiation, consistent with our previously reported results [30].

**Fig 4:**
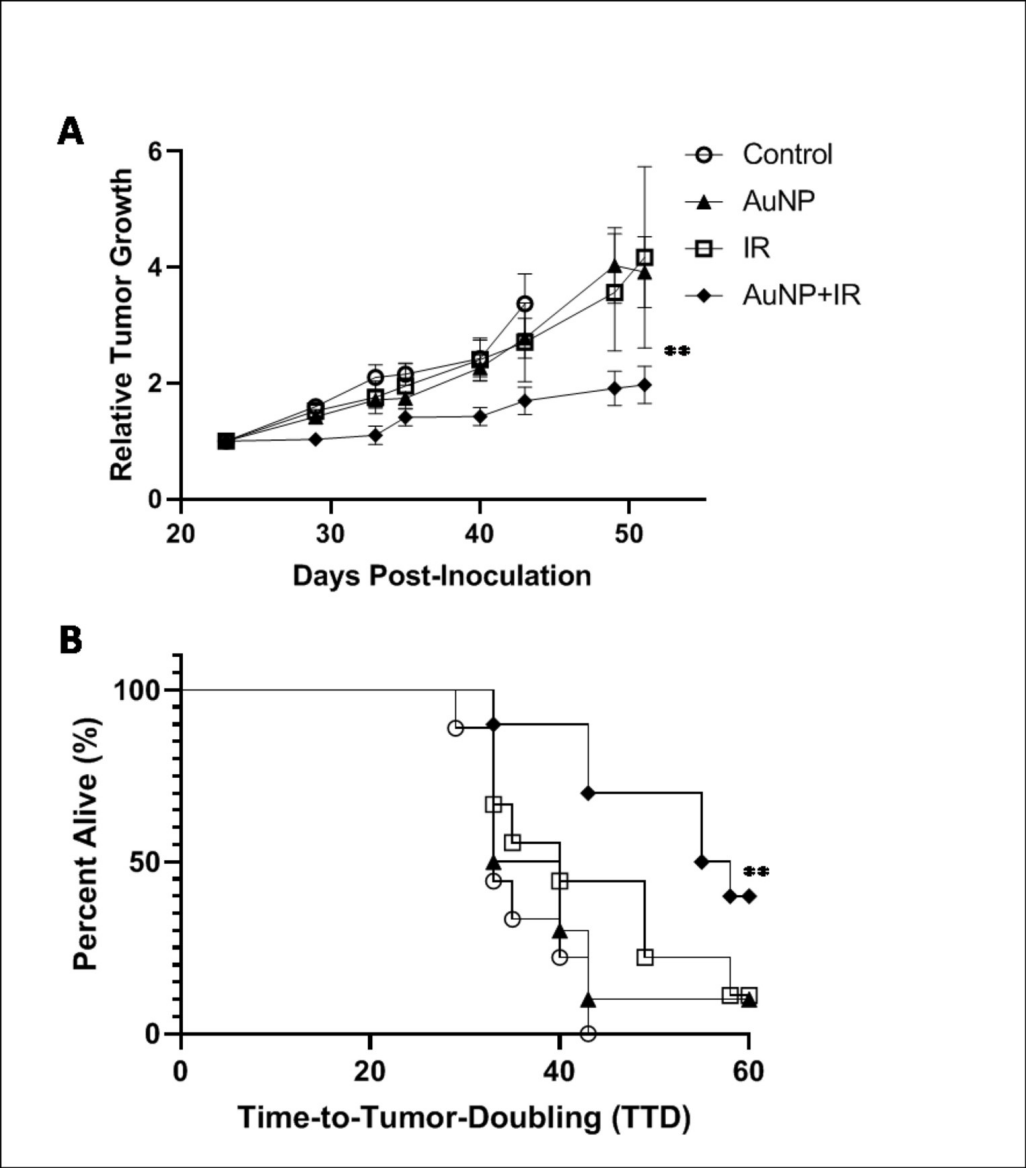
Therapeutic evaluation of tumor vascular disruption. (A) Tumor growth relative to tumor size on day of irradiation is shown. A significant (**P<0.*05*) delay in tumor growth was observed for AuNP+IR treated mice compared to the controls up to 50 days (n = 9–10). Values represent mean growth ± SD. (B) Time-to-Tumor-Doubling (TTD) is shown with endpoint failure defined as tumor volume doubling relative to day of treatment. A significant (**P<0.*05*) delay in doubling, which is an indicator of a progressive disease, is seen for mice treated with AuNP+IR compared to all control groups (n = 9–10).

Evaluation of the therapeutic benefit demonstrated significant (*P<0*.*05*) tumor growth suppression as a result of the combined anti-vascular approach. There was no statistical difference between tumor growth in the control (no therapy) and AuNP or radiation only treatment groups (Fig 4A). The relative tumor growth 50 days post-inoculation was substantially reduced in the AuNP+IR treated group compared to all control groups. Due to tumor ulceration occurring in all cohorts (including no treatment) necessitating pre-emptive euthanasia, Time-to-Tumor-Doubling (TTD) was accessed relative to day of treatment as a determinant of a progressive disease. As seen in Fig 4B, mice treated with AuNP+IR (56.5 d) had significantly (*P<0*.*05*) delayed TTD compared to all control groups (33, 36.5, and 40 d for Control, AuNP, and IR, respectively). Given the trend in the tumor growth curves and TTD, one would reasonably expect the AuNP+IR treated group to exhibit long-term survival. Mice with no tumor ulcerations in the AuNP+IR group survived over 110 days post-inoculation, with minimum change in tumor volume indicative of long-term tumor growth delay. This was further observed in a previous study using the same nanoparticle formulation and radiation regiment in a mouse model of pancreatic cancer [31].

**Fig 5:**
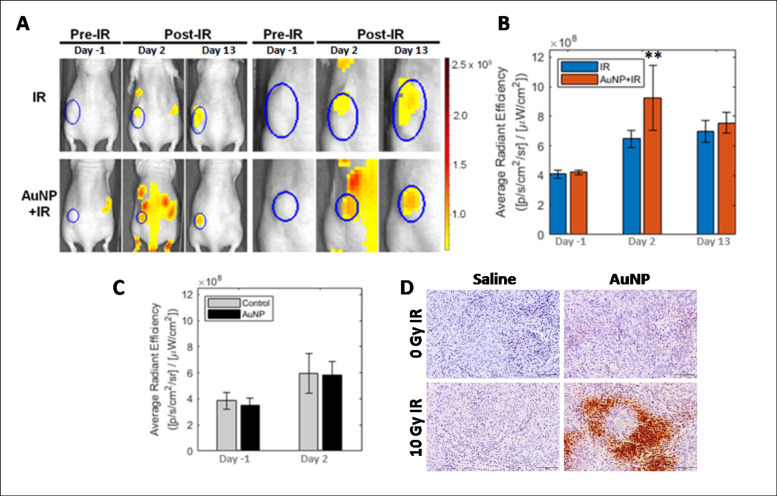
Measuring therapy-induced tumor hypoxia in A549 non-small cell lung cancer xenografts. (A) Fluorescence imaging of tumor hypoxia in representative mice from the IR-only and AuNP+IR groups with A549 xenografts. Mice were injected with HypoxiSense680 48 h before imaging. Tumor hypoxia is visualized pre- and post-10 Gy irradiation treatment. Representative images show the whole mouse and a magnified image of each tumor ROI. The color bar scale shows average radiant efficiency. (B) Plots show the mean quantification of each tumor ROI (Radiation Efficiency ([photon/sec]/μWatt/cm^2^)) on each day of imaging (Day -1, 2 and 13). In the IR-only group, the images show a 1.5-fold increase in signal 48 h post-IR compared to baseline while 11 days later the tumor hypoxia remained stable. The AuNP+IR group showed a 2.5-fold increase (**P<0.*05*) at 48 h post-IR and then a decrease to the level of IR-only signal 11 days later. Data presented as mean ± SD (n = 3). (C) The relative radiation efficiency intensity of HypoxiSense680 is similar in the control and GNP-only groups, indicating no interference from the attached fluorophore. (D) Qualitative histological assessment of tumor hypoxia by pimonidazole staining further confirmed the increase in the hypoxia following tumor vascular disruption at 48 h. Scale bar = 100 μm.

We measured tumor hypoxia pre- and post-tumor vascular disruption at early and late time points. HypoxiSense680, a fluorescent probe that binds to carbonic anhydrase (CAIX) that is upregulated in hypoxic regions especially in non-small cell lung cancer [46], was utilized following the manufacturer’s protocols. Mice were imaged 48 h post-injection to minimize noise from any unbound or non-specifically accumulated probe and maximize tumor hypoxic region uptake and retention. Whole body FLI showed a nearly 2.5-fold increase in HypoxiSense680 signal at 48 h post-tumor vascular disruption for the AuNP+IR group (Fig 5A and 5B). However, in the IR-only group, this increase was substantially smaller compared to the non-treated controls. A comparison of the control and AuNP-only groups indicated that the AuNP contributed no additional fluorescence signal (Fig 5C). Measured increases in these control groups are likely due to the continued tumor growth. These findings were confirmed with representative pimonidazole staining that showed an increase in tumor hypoxia 48 h after AuNP+IR treatment (Fig 5D).

## Discussion

This is the first study to provide evidence that vascular targeted AuNPs combined with radiation causes a transient increase in tumor hypoxia but still results in better tumor control compared to radiation-alone treatment. Our experimental findings suggest that vascular targeted AuNPs combined with 10 Gy clinical 6 MV irradiation temporarily increased tumor hypoxia compared to control groups as confirmed by *in vivo* fluorescence imaging and immunohistochemistry. Multiple studies have evaluated the role of radiation dose in either tumor cell or endothelial cell death as related to overall growth inhibition [47–49]. Studies have confirmed that low doses of radiation which are conventionally used in fractionated radiotherapy (2–5 Gy) only cause temporary changes to tumor vasculature, however doses > 8 Gy lead to endothelial cell death and vascular disruption [47–50]. Previous work in our lab has confirmed that a single dose of 10 Gy radiation increases vascular damage and permeation, which is further amplified with the addition of AuNPs [31, 51]. A study to evaluate the change in tumor perfusion in a human laryngeal squamous cell tumor indirectly correlated with the results presented of a decrease in perfusion post irradiation with a single dose of 10 Gy followed by some recovery in 7–11 days [52]. Previous work by Fuks and Kolesnick reported a potential mechanism for endothelial cell damage via the ceramide mediated apoptosis pathway that was increased at radiation doses of 8–10 Gy but no identifiable pathway changes < 3 Gy [53]. Our results indicating changes in hypoxia due to vascular disruption with a single dose of 10 Gy radiation and AuNP aligns with previous works confirming high doses of irradiation leads to transient changes in tumor endothelial cells which are not seen in conventionally used < 2Gy fractionated therapy [47, 50].

Radiation therapy is dependent on the oxygen enhancement ratio (OER) which refers to the enhancement of ionizing radiation in the presence of oxygen [54]. Currently studied VDAs can hinder the efficacy of radiation therapy by reducing tumor oxygenation over a prolonged period and migrating tumors towards a more aggressive phenotype. Here we have shown that although the underlying mechanism of treatment is still vascular disruption, overall tumor growth was still hindered compared to control treatment and long term hypoxia was prevented. This transient vascular disruption was further confirmed by Kwon et al. demonstrating an increase in microbubble perfusion 24 hours post therapy followed by normalization to baseline levels, indicative of normalization of tumor vasculature flow [55].

The negative impact of hypoxia on radiation therapy has been known for decades [56, 57]. Fractionated radiation therapy takes advantage of time between doses to balance reoxygenation of cells versus repopulation of surviving cells to improve the efficacy of therapy [57, 58]. Tissue that is either hypoxic or anoxic during radiation are up to three times more likely to be radioresistant compared to oxygenated tissue [59]. A recent study compared 10–30 Gy radiation doses to vascular damage and microenvironment reoxygenation [57]. Similar to the work presented, they showed an increase in hypoxia markers, including HIF-1α, pimonidazole, and carbonic anhydrase 9, post-radiation followed by a decrease around 10 days post 20 Gy radiation [57]. They further showed that tumor reoxygenation was dose dependent and higher doses of radiation led to a rapid increase in hypoxic cells followed by reoxygenation of surviving fractions [57]. This transient change in reoxygenation is crucial for conventionally use fractionation approaches and treatment planning. In the work presented here, we observed tumor growth suppression by combining targeted nanoparticles with radiation therapy, despite the transient increase in induced hypoxia, indicating that this could be a potential clinical strategy. A recent study compared the impact of incorporating tumor hypoxia measurements in NSCLC patient prescribed dose calculations and identified the increased importance of hypoxic fractions within a tumor relative to overall tumor volume [56].

In summary, we have found that tumor vascular disruption due to the vascular targeting and radiation dose amplification properties of AuNPs resulted in a short-term transient increase in tumor hypoxia as well as long-term tumor growth suppression. Histology demonstrated no damage to normal tissues as a result of the therapy. As with many other anti-angiogenic or anti-vascular treatments, there are issues that still need to be addressed related to the optimal timing of subsequent therapies (radiation and/or chemotherapy) in order to minimize the impact of the induced hypoxia. Non-invasive molecular imaging techniques such as functional MRI, specifically blood oxygen level dependent (BOLD) MRI to continuously monitor hypoxia or dynamic contrast enhanced (DCE) MRI to monitor vascular permeability [60–63], photoacoustic imaging to monitor changes in hemodynamic characteristics [64], or more conventionally used positron emission tomography (PET) [65]could be used to further investigate earlier time points to resolve the kinetics of tumor hypoxia after vascular targeted AuNP and radiation therapy as well as determine an optimal dose schedule to maximize the OER during radiation therapy. In addition, future work incorporating an orthotopic NSCLC model can be studied to understand the microenvironment changes expected post-vascular disruption in a more clinically representative model. Recent studies comparing subcutaneous and orthotopic lung cancer models confirm the improved vascular functionality and in turn reduced hypoxia within orthotopic models [66, 67]. In this study we have shown that regardless of a transient hypoxic tumor microenvironment, tumor burden significantly decreased following vascular disruption therapy with targeted AuNP and IR. The increased vascularity of orthotopic tumors may improve AuNP distribution, subsequently further increasing the impact of IR. In addition, the expected lower hypoxia in orthotopic tumors would increase the OER effect. Therefore, this therapy is expected to have a stronger impact in orthotopic versus subcutaneous NSCLC models. The current study is the first step towards identifying a unique transient hypoxic tumor environment post-vascular disruption therapy, however further work with continuous monitoring techniques could reveal opportunities to further improve radiation therapy outcomes.

## Supporting information

S1 Fig Representative AF647 and HS680 spectra. Representative spectra showing excitation and emission spectra of AF647 and HS680. The excitation and emission were chosen specifically to minimize signal from AF647 when evaluating HS680 hypoxia florescence.
(TIF)
