# Supplementary figures and images for: Noninvasive imaging of tumor hypoxia after nanoparticle-mediated tumor vascular disruption
Source: PLoS One. 2020 Jul 24;15(7):e0236245. doi: 10.1371/journal.pone.0236245 (PMC7380644; doi:10.1371/journal.pone.0236245)

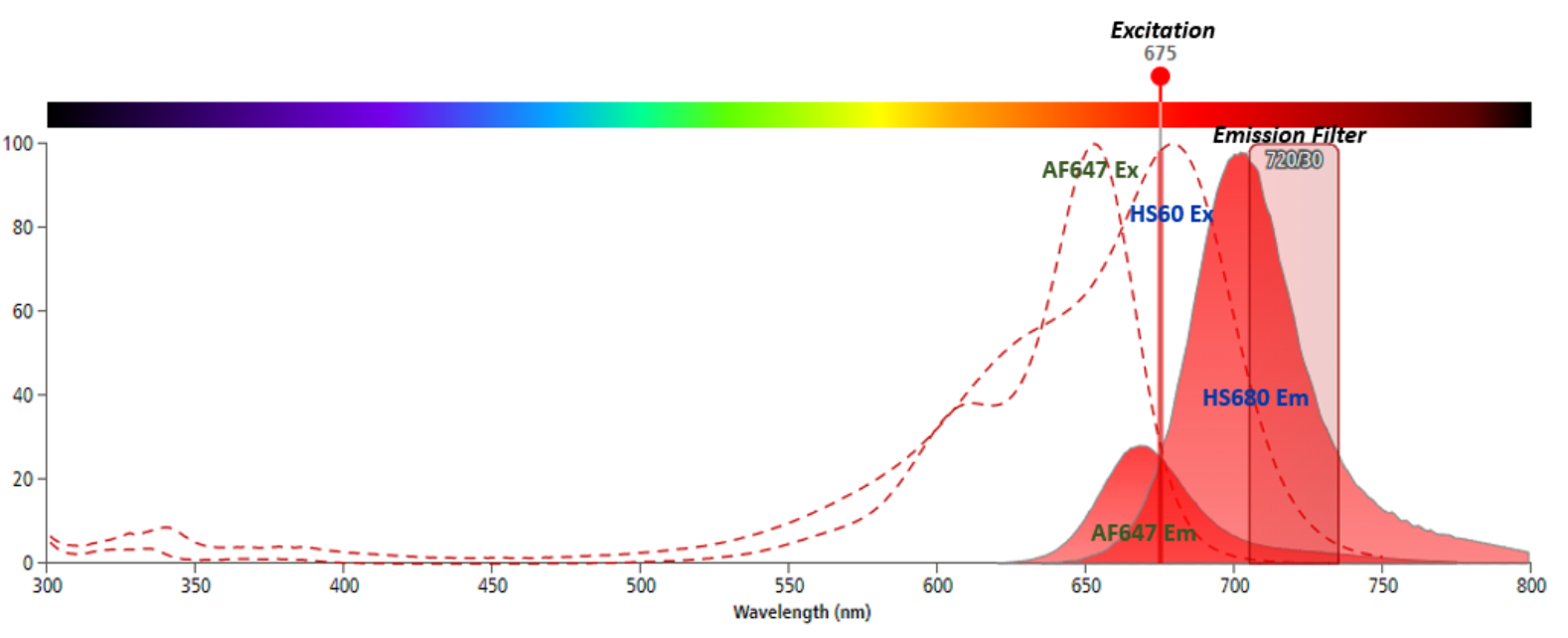

Supplement: S1 Fig — Representative spectra showing excitation and emission spectra of AF647 and HS680. The excitation and emission were chosen specifically to minimize signal from AF647 when evaluating HS680 hypoxia florescence. (TIF) [file pone.0236245.s001.tif]
